# Tissue Distribution of *trans*-Resveratrol and Its Metabolites after Oral Administration in Human Eyes

**DOI:** 10.1155/2017/4052094

**Published:** 2017-03-20

**Authors:** Shuaishuai Wang, Zheng Wang, Shuo Yang, Tiemei Yin, Yaoli Zhang, Yuanjun Qin, Robert N. Weinreb, Xufang Sun

**Affiliations:** ^1^Department of Ophthalmology, Tongji Hospital, Tongji Medical College, Huazhong University of Science and Technology, 1095 Jie-fang Road, Wuhan, Hubei Province, China; ^2^Department of Ophthalmology, General Hospital of Wanbei Coal Group, Suzhou, Anhui 234000, China; ^3^School of Chemistry and Engineering, Wuhan University of Technology, Wuhan 430070, China; ^4^Eye Center, Second Affiliated Hospital, School of Medicine, Zhejiang University, Hangzhou 310000, China; ^5^Shiley Eye Institute and Department of Ophthalmology, University of California, San Diego, La Jolla, San Diego, CA, USA

## Abstract

*Purpose.* This study was performed to measure the concentration of *trans*-resveratrol and its three metabolites in human eyes. *Methods.* The patients who underwent pars plana vitrectomy for rhegmatogenous retinal detachment were included. The participants were orally given *trans*-resveratrol-based supplement (Longevinex®). A suitable amount of conjunctiva, aqueous humor, and vitreous humor were obtained during the operation. High-performance liquid chromatography (HPLC) with mass spectrometry (LC/MS/MS) was used to detect the concentration of *trans*-resveratrol and its three metabolites in the various samples. *Results.* The average concentration of resveratrol in the conjunctiva was 17.19 ± 15.32 nmol/g (mean ± SD). The concentration of resveratrol in the aqueous humor was close to the limit of detection, but its metabolites could be quantified. The concentrations of resveratrol metabolites in the aqueous humor can be detected. In the vitreous humor, the average concentration of resveratrol-3-O-sulfate was 62.95 ± 41.97 nmol/L. The sulfate conjugations of resveratrol were recovered in the conjunctiva, aqueous humor, and vitreous humor. *Conclusions.* Resveratrol and its three metabolites can be detected in the ocular tissues after oral administration. Although the concentration of parent resveratrol was low in the eyes, its metabolites could be detected and may have a role in the treatment of ocular diseases.

## 1. Introduction

**Figure 1:**
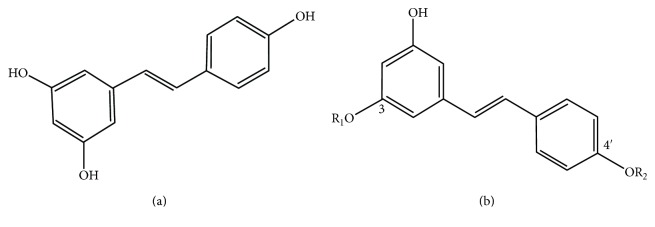
(a) Chemical structure of *trans*-resveratrol. (b) The structure of the metabolites of resveratrol: (1) R_1_ = sulfate, R_2_ = H: resveratrol-3-O-sulfate, (2) R_1_ = H, R_2_ = glucuronide: resveratrol-4′-O-glucuronide, and (3) R_1_ = glucuronide, R_2_ = H: resveratrol-3-O-glucuronide.

Resveratrol (3,4′,5-trihydroxystilbene) is a natural polyphenolic phytoalexin that is mainly found in grapes, leading to its high concentration in wine [1]. Additional sources include peanuts and cranberries [2]. Resveratrol has a stilbene structure, and the compound consists of two aromatic rings connected by a methylene bridge [3] (Figure 1). Interest has arisen due to its cardioprotective action on the vasculature, especially its ability to inhibit angiogenesis and to facilitate vasorelaxation [4]. Resveratrol has been suggested to promote health in dry eye, glaucoma, cataracts, age-related macular degeneration (AMD), and diabetic retinopathy (DR). Resveratrol increases blood perfusion, has anti-inflammatory effects, and reduces oxidative stress and angiogenesis [3]. However, it has been unclear whether it can reach the posterior segment of the eye.

Studies performed on rats have shown that resveratrol is rapidly metabolized in the gut prior to entering the systemic circulation [5, 6]. The reduced concentration of resveratrol entering the systemic circulation is compounded further by recirculation between the small intestine and the liver. Metabolism occurs so rapidly that a conjugate of resveratrol has been detected by high-performance liquid chromatography (HPLC) less than 10 minutes following intravenous infusion in human subjects [7]. It has been hypothesized, however, that despite the fact that the *trans*-resveratrol glucuronides have been shown to be inactive in vitro, they may be active in vivo, and the sulfate conjugate serves as an inactive pool for resveratrol [8, 9]. Prior studies have assessed the concentration of resveratrol in animal and human plasma [10–13]. However, an understanding of the concentration of resveratrol in tissues, especially in human eyes, is lacking. Several studies have reported the pharmacokinetics and tissue distribution of resveratrol in the liver, kidneys, lungs, and heart [14, 15]. These studies were mostly based on animal models; few researchers have used human subjects for experimentation.

The aim of the present study was to investigate the tissue distribution of parent resveratrol and its main three metabolites in human eyes after oral administration. This study also provides information about the activity of the metabolites [8].

## 2. Materials and Methods

### 2.1. Patients

The eligible participants were inpatients undergoing pars plana vitrectomy with retinal detachment repair at Tongji Hospital, Tongji Medical College. Their health status was determined by an interview, medical history, physical examination, 12-lead electrocardiogram (ECG), and clinical laboratory testing. All participants also underwent a detailed ophthalmic examination, including best-corrected visual acuity (BCVA) (LogMAR), intraocular pressure (IOP), and fundus examination. All of the eyes had been diagnosed just before being consented with a rhegmatogenous retinal detachment. There were no obvious vitreous cavities and no anterior chamber bleeding. Female subjects who were pregnant, patients with anemia, and those who had taken any medication within one week prior to admission were excluded from participation. Alcohol and cigarettes were forbidden from 48 hours before admission until discharge from the clinic. The participants were advised to avoid consumption of coffee, tea, or cocoa-containing foods during the study. No foods rich in resveratrol (e.g., grapes, peanuts, and their products) were allowed until discharge.

The study protocol was approved by the Huazhong University of Science and Technology Institutional Review Board and Ethics Committee. The study was conducted according to the principles of the Declaration of Helsinki, and all participants gave their written, informed consent prior to participation. This study is registered at ClinicalTrials.gov (registration number: NCT02321176; registration date: July 2014).

### 2.2. Intervention

Longevinex (Resveratrol Partners LLC, Las Vegas, NV), a nutritional supplement, has 100 mg of *trans*-resveratrol (purity 85–92%). The micro-sized powder also contains stabilized 25 mg quercetin (purity 98%), 75 mg rice bran (70% extract IP6, 30% calcium phytate), vitamin D_3_ (1000 IU), and 10 mg nucleotides (for DNA repair). *trans*-Resveratrol is the active form of resveratrol. The other four substances have a synergy function in mediating the bioavailability of resveratrol. Micronization enfolded in plant starches and dextrins to protect from light, heat, and oxygen exposure. This blend has been demonstrated to obtain synergism in studies conducted at two academic centers and the National Institutes of Health in the United States [16]. Each of the participants was given one capsule orally daily for a total of three doses prior to tissue collection. The capsules were taken in the morning between 6:00 a.m. and 7:00 a.m. The last dose was administered in the morning before surgical resection.

### 2.3. Sample Preparation

The surgical resection was performed approximately 2–4 hours after the final dose of resveratrol. The conjunctivas were collected, placed on ice, and protected from light. Frozen tissue samples were mixed with liquid nitrogen and ground, then weighed and homogenized with methanol. The samples were then snap-frozen and kept at −80°C until analysis. Aqueous humor and vitreous humor were collected in dry EP tubes at the time of resection. The tubes were chilled on ice, protected from light, and stored at −80°C until analysis, which was performed within 6 months. We get the tissue sample weight or volume: conjunctiva (2.8 mg–13.7 mg), aqueous humor (50 *μ*L–100 *μ*L), and vitreous humor (about 200 *μ*L).

Venous blood was collected in lithium heparin tubes at the time of resection (about 2 hours after the final dose of resveratrol). Tubes were chilled on ice and protected from light. Plasma was kept at −80°C until analysis, which was also performed within 6 months. The volume of the blood withdrawn was about 2 mL.

### 2.4. Analysis of Resveratrol and Its Metabolites

All of the samples were extracted with a precipitant (methanol : acetonitrile = 1 : 1), then vortexed for 1 min. Liquid samples were centrifuged (3000 ×g, 4°C, 15 min). The supernatant was removed and analyzed by liquid chromatography tandem mass spectrometry (LC/MS/MS) with multiple reaction monitoring (MRM), operated in the negative ion mode as described previously [17]. The chromatographic column was a Welch Ultimate (2.1 mm × 50 mm, 5 *μ*m), and the guard column was a Phenomenex C18 (4 mm × 3.0 mm, 5 *μ*m). The parent resveratrol and metabolites were separated by using the mobile phase consisting of acetonitrile containing 0.1% methanoic acid and pure water with a flow rate of 0.3 mL/min. The quantitation of resveratrol and its three metabolites using a gradient HPLC system (Shimadzu, LC–30AT) coupled with MS/MS system (ABsciex Qtrap4500) was performed using standard materials and a method that had previously been validated in terms of interday and intraday variability, recovery, accuracy, and precision [17]. The limit of quantitation was 5 ng/mL, whereas the limit of detection was half of these values. The resveratrol and its metabolites in biomatrices were stable under the storage and assay conditions. The standard material of the parent resveratrol, resveratrol-3-O-sulfate, resveratrol-3-O-glucuronide, and resveratrol-4′-O-glucuronide were provided by Santa Cruz Biotechnology, Inc. (Santa Cruz, CA, USA). The internal standard was carbamazepine. All of the samples were extracted and analyzed in duplicate, and the mean value was used. The statistical analysis was performed by using SPSS 16.0. All the variables were demonstrated as arithmetic means.

## 3. Results

### 3.1. Characteristics of the Participants

20 men and 15 women, with a mean age of 51.17 ± 14.53 years (range 20–74 years), were included. There were a total of 35 eyes (left/right = 19/16). The mean intraocular pressure was 11.89 ± 2.87 mmHg (range 5.0–20 mmHg). The BCVA (LogMAR) in the study group was in the range of LP–1.3 (Table 1).

### 3.2. Identification of Resveratrol and Its Metabolites in Eye Tissues

**Figure 2:**
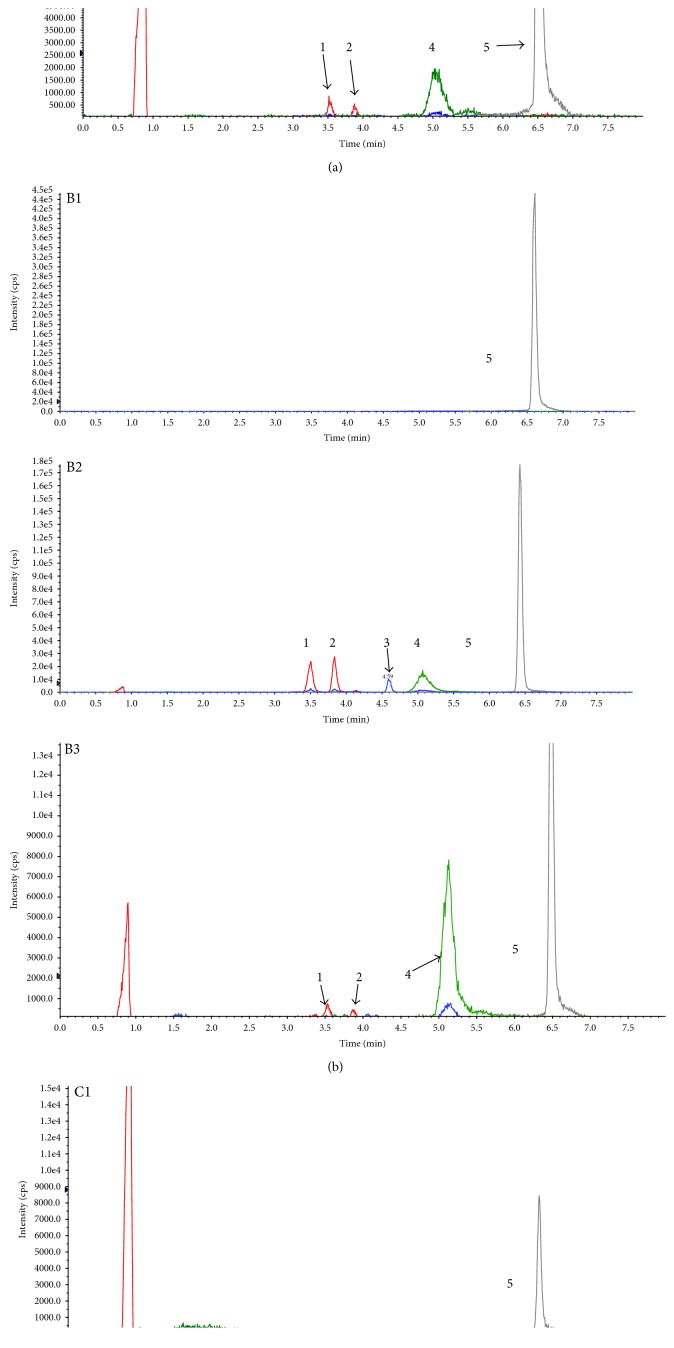
(A1) LC-MS chromatography of the aqueous humor of one patient who did not receive oral resveratrol. (A2) LC-MS chromatography of standard solution of resveratrol, its major three metabolites, and carbamazepine (internal standard). (A3) LC-MS chromatography of the aqueous humor of one patient who had taken one capsule daily for three days before tissue extraction. (B1) LC-MS chromatography of the vitreous humor of one patient who did not receive oral resveratrol. (B2) LC-MS chromatography of standard solution of resveratrol, its three major metabolites, and carbamazepine (internal standard). (B3) LC-MS chromatography of the vitreous humor of one patient who had taken one capsule daily for three days before tissue extraction. (C1) LC-MS chromatography of the conjunctiva of one patient who did not receive oral resveratrol. (C2) LC-MS chromatography of standard solution of resveratrol, its three major metabolites, and carbamazepine (internal standard). (C3) LC-MS chromatography of the conjunctiva of one patient who had taken one capsule daily for three days. 1: resveratrol-4′-O-glucuronide; 2: resveratrol-3-O-glucuronide; 3: resveratrol; 4: resveratrol-3-O-sulfate; and 5: carbamazepine.

**Figure 3:**
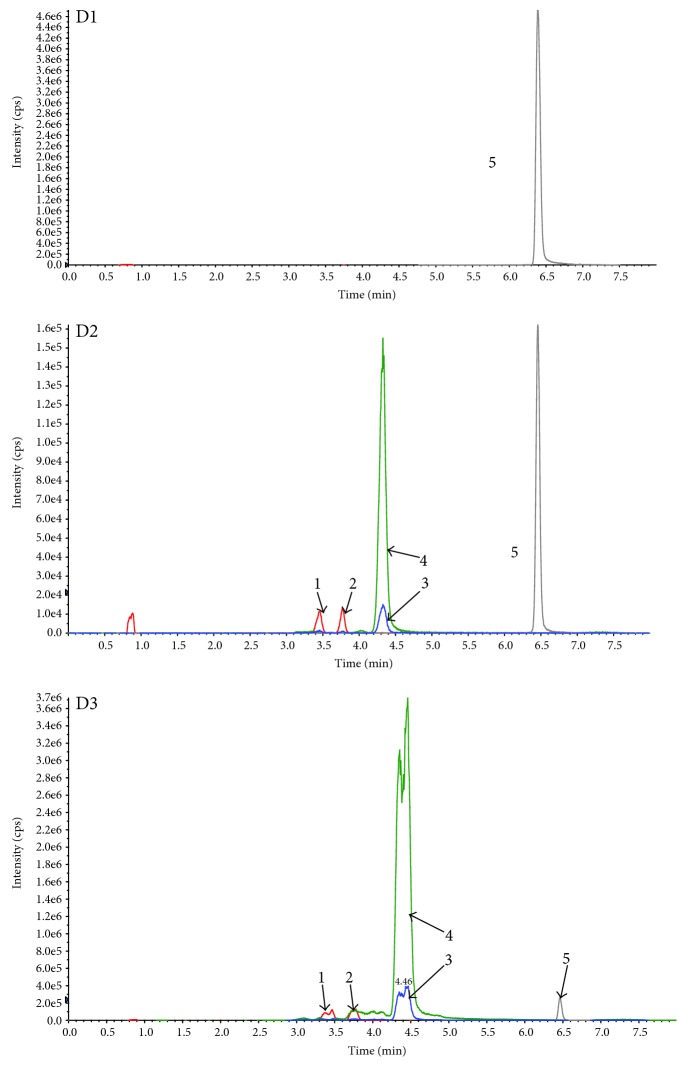
(D1) LC-MS chromatography of the serum of one patient who did not receive oral resveratrol. (D2) LC-MS chromatography of standard solution of resveratrol, its major three metabolites, and carbamazepine (internal standard). (D3) LC-MS chromatography of the serum of one patient who had taken one capsule daily for three days before blood extraction.

**Figure 4:**
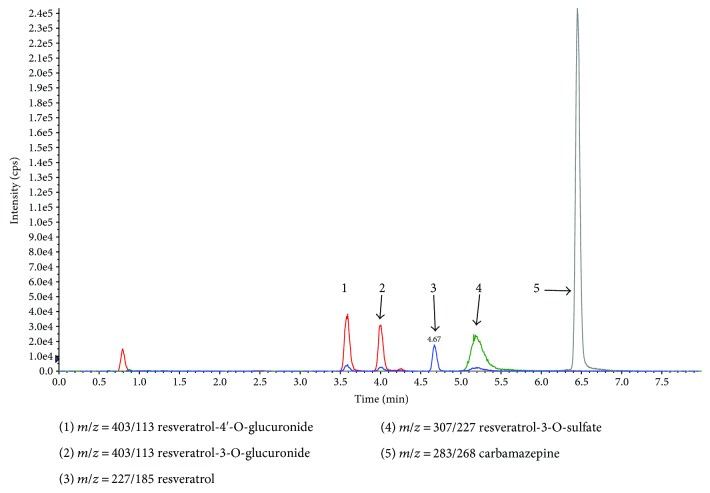
The chromatograms and the related mass-to-charge ratio (*m*/*z*) of the total materials using chromatography combined mass spectrometry.

Resveratrol and its metabolites were recovered from human eye tissues and identified by HPLC/MS/MS analysis with authentic reference material.  Figure 2 shows three groups of representative HPLC data from the tissue extracts of three patients who had ingested three doses of resveratrol, compared with chromatograms from three patients who did not receive the experimental capsule and had no detectable resveratrol or its metabolites. We have detected the blood of three patients of the participants. And the parent resveratrol and its metabolites could be quantified (Figure 3). Identity was confirmed by suitable MRM mass transitions of mass-to-charge ratios (*m*/*z*). The following species could be identified by mass spectrometry (Figure 4): *trans*-resveratrol (*m*/*z*: 227/185), resveratrol-3-O-glucuronide (*m*/*z*: 403/227), resveratrol-4′-O-glucuronide (*m*/*z*: 403/227), resveratrol-3-O-sulfate (*m*/*z*: 307/227), and carbamazepine (*m*/*z*: 283/268).

### 3.3. Concentrations of Resveratrol and Its Metabolites in Eye Tissues

The parent resveratrol and its three metabolites were quantitated in the participants' eye tissues (Table 2). In most tissues, the concentrations of resveratrol's metabolic conjugates were higher than those of the parent resveratrol. Resveratrol or its metabolites was not detected in 10 out of 35 eyes. In at least the conjunctival samples of 25 eyes, we could detect resveratrol or its metabolites. The concentrations of resveratrol and its metabolites demonstrated inter- and intraindividual variability. In the conjunctiva, the parent resveratrol metabolites were quantified in some samples and eight samples were quantified for the parent resveratrol. The mean concentration was 17.19 ± 15.32 nmol/g. The mean tissue concentrations determined for resveratrol metabolites were 2.32 ± 2.00 nmol/g for resveratrol-3-O-glucuronide in eight samples, 2.12 ± 2.09 nmol/g for resveratrol-4′-O-glucuronide in nine samples, and 22.31 ± 24.18 nmol/g for resveratrol-3-O-sulfate in 17 samples. In the aqueous humor, the peak of the parent resveratrol was very low and the signal-to-noise ratio (SNR) was <3, but the retention time of the parent resveratrol in the samples conformed to the retention time of its standard curve. Therefore, we assumed that there was a slight amount of the parent resveratrol in the aqueous humor. The three major metabolites could also be quantified in the aqueous humor, with mean concentrations of resveratrol-3-O-glucuronide, resveratrol-4′-O-glucuronide, and resveratrol-3-O-sulfate of 86.11 ± 30.76 nmol/L (*n* = 3), 85.17 ± 56.73 nmol/L (*n* = 4), and 364.9 ± 523.94 nmol/L (*n* = 7), respectively. In the vitreous humor, we only quantified resveratrol-3-O-sulfate, which had a mean concentration of 62.95 ± 41.97 nmol/L (*n* = 6). The SNRs of the parent resveratrol, resveratrol-3-O-glucuronide, and resveratrol-4′-O-glucuronide were <3, so their true values were below the limit of detection. However, all of the retention times of the materials conformed to their standard carves, indicating that there was also a slight amount of the parent resveratrol-3-O-glucuronide and resveratrol-4′-O-glucuronide in the vitreous humor.

### 3.4. Concentrations of Resveratrol and Its Metabolites in Plasma

Plasma was obtained from three patients of the participants at the point of surgery and analyzed by LC/MS/MS. Resveratrol and its metabolites were at quantifiable concentrations (Table 3). The highest level (1969.5 nmol/L) was observed for resveratrol-3-O-sulfate in the three patients.

## 4. Discussion

This study is the first to detect resveratrol and its three metabolites in human eyes after oral administration of this compound. Longevinex showed synergistic effects of these compounds. Schlachterman et al. [18] reported a combination of resveratrol, quercetin, and catechin at different concentrations where each significantly reduced cell proliferation and blocked cell cycle progression in vitro. Also, other article reported when polyphenols were fractionated and isolated, the benefits of the whole extract were greater than the sum of its parts, which indicated a synergism [19].

The results of this study showed that neither resveratrol nor its metabolites were detected in 10 out of 35 eyes. Resveratrol and its metabolites could be detected in at least the conjunctiva of 25 eyes. The concentrations of resveratrol and its metabolites showed inter- and intraindividual variability. This feature is in line with previous in vivo studies [17]. In our study, at least one of the three metabolites could be quantified in the three different tissues. It is clear that the concentrations also depend on the size and volume of the tissues. The time span for obtaining samples was also different, and this may also be a cause of the different concentrations in different tissues. However, the real evaluation of the concentration of resveratrol in vivo is difficult. Resveratrol could combine with lipoproteins or cell membranes, so it appears to have low bioavailability.

The blood-ocular barrier contains the blood-retinal barrier (BRB) and the blood-aqueous humor barrier (BAB). The BAB is formed by endothelial cells of the blood vessels in the iris and the nonpigmented cell layer of the ciliary epithelium. The BRB is composed of retinal pigment epithelium (RPE) and the tight retinal capillary wall. One study reported that the BAB is less functional than the BRB in human eyes and that the permeability of the BAB to hydrophilic substances is higher than that of the BRB [20]. Tsuboi et al. [21] reported a significant increase in permeability across the RPE in detached eyes with retinal tears. A similar situation was reported by Cantrill and Pederson [22] in monkey eyes with detached retinas. Retinal tears increase the permeability of the BRB because of damage to the local physical structure of the neurosensory retina, although the RPE layer is still intact. The release of factors can influence local vascular permeability in the anterior segment. Little et al. reported that blood vessels in the iris and ciliary body of patients with rhegmatogenous retinal detachment are much more permeable than those of normal controls [23]. In our experimental results, the hydrophilic substances of resveratrol metabolites were the main quantitative substances in the aqueous humor and the vitreous humor. The most reasonable explanation for this is that the resveratrol was quickly metabolized into its three metabolites when orally administered. The metabolites then entered the aqueous humor and vitreous humor, especially the aqueous humor. Resveratrol and its metabolites were higher in the conjunctiva. This may be the reason for little residual blood in the conjunctival vessels. This result could provide experimental support for the treatment of ocular surface diseases [24]. The concentration of the parent resveratrol and its three metabolites in the serum was higher than that of the ocular tissues at an equivalent time point. Resveratrol is firstly aborbed in the intestine and then enters the systematic circulation and finally enters the eye. And the blood can provide drugs for the eyes continuously.

### 4.1. Possible Biological Functions Associated with the Concentration of Resveratrol

In our study, the concentration of resveratrol and its metabolites was measured at the nM level (nmol/L). The parent resveratrol could be quantified only in the conjunctiva. The highest and average concentrations were 45.12 nmol/g and 17.19 nmol/g, respectively. In the aqueous humor and vitreous humor, the free resveratrol was below or close to the limit of the detectable level at 10.96 nmol/L. The literature reveals that the concentration of resveratrol in red wine is variable; for example, the level in a moderate amount of red wine (300–600 ml) is ~2.4 nM and the concentration of the total equivalents is ~180 nM [25]. The long-term drinking of red wine is considered beneficial for the human body because, in part, it contains resveratrol; this has been demonstrated epidemiologically and in in vitro and in vivo experiments. The protective effect is established on the basis of dietary concentrations, which are always very low in vivo.

Pearce et al. found that resveratrol could activate telomerases at a concentration of 10^−8^ M and could immortalize mammary epithelial progenitors [26]. However, interestingly, this only occurs at the lowest concentration. When the concentration is increased at the micromolar level of resveratrol in certain cellular models, it may upregulate p53 by increasing the cellular content and inducing posttranslational modifications, resulting in senescence and apoptosis [26]. In contrast, Waite et al. found that 0.1 nM resveratrol could activate the PTEN protein to inhibit the proliferation of breast cancer cells [27]. Another study reported that nanomolar concentrations of resveratrol have cardiovascular protective effects, including inhibition of platelet aggregation [28]. Resveratrol exhibits a U- or J-shaped risk curve [16]. In conclusion, some experiments have reported opposite results and further clinical research is needed to explore the optimal concentration of resveratrol for the treatment of human disease.

There only have been few studies to detect the concentration of resveratrol in human tissues. Some reported that resveratrol accumulates in the bile, stomach, liver, and kidneys [29]. Many experiments have been done in vitro or by using animal models associated with the eyes. Micromolar concentrations of resveratrol inhibit the proliferation and migration of blood vessel endothelial cells that are crucial for angiogenesis [30, 31], protect against lens epithelial cell apoptosis in diabetic cataracts in rats [32], protect against inflammatory ocular surface diseases, such as dry eye disease and severe allergies [24], and inhibit the proliferation of retinoblastoma cells [3]. The nanomolar concentration of resveratrol necessary for effective treatment of the human eyes has not been demonstrated. Clinical drug trials are more complicated than animal or in vitro experiments, as they require more time, even years, for a treatment cycle. Many reports have proved that resveratrol is beneficial to the human eyes [16, 33]. This effect may be due to the long-term or short-term effects associated with the expansion of blood vessels in the eyes [34, 35], and other compounds in the drug may have a synergistic effect with resveratrol. One article reported that quercetin could inhibit resveratrol sulfate in the liver and duodenum [36], and it could also enhance the resveratrol-induced reduction of inflammation and oxidative stress in ocular surface epithelial cells [24]. The Longevinex that we used in this experiment was described in detail in our previous study [35]. Its main ingredients are *trans*-resveratrol, quercetin, vitamin D, and ferulic acid.

### 4.2. Concentration of Resveratrol's Metabolites and Physiological Significance In Vivo

In this study, the main metabolite in the eye tissues was resveratrol-3-O-sulfate. In the conjunctiva, aqueous humor, and vitreous humor, the average concentrations of this metabolite were 22.31 nmol/g, 364.9 nmol/L, and 62.95 nmol/L, respectively. This is in accordance with previous reports that resveratrol has a short half-life (~8–14 min) in the body and is then widely metabolized [37, 38]. However, one study reported that parent resveratrol was the main form in the organs and tissues [39]. In our experiment, the metabolites, especially resveratrol-3-O-sulfate, were the main form in eye tissues. Overall, these findings contrast with results obtained previously [39]. Schueller et al. reported that sulfated metabolites of the parent resveratrol showed a beneficial potential for attenuating inflammatory immune processes [40]. It is also important to note that resveratrol metabolites were thought of as the pool of the parent resveratrol [8], so there was an assumption that the resveratrol metabolites may perform certain activities or transform into parent resveratrol to have a beneficial effect in organs and tissues. With only three days of administration, reservatrol and its metabolites could still be detected throughout the eye.

Many articles provide evidence that resveratrol's biological activities include vasorelaxant activity, antiangiogenesis activity, anti-inflammatory activity, antioxidant activity, and so on through different molecular mechanisms using animal models and in vitro retinal cells [3, 25, 41]. It is clear that resveratrol may have potential in the treatment of several ocular diseases, such as primary open-angle glaucoma (POAG) [42], age-related macular degeneration (AMD) [3], diabetic retinopathy (DR) [3], and even ocular tumors [43]. In light of the current interest in resveratrol as a potential preventive and therapeutic agent, we hope that our results, measuring resveratrol levels and its metabolites in the human eye, will help define doses that may be used in future treatment and in the prevention of ocular disease.

## Figures and Tables

**Table 1:** Characteristics of patients who were recruited into the study.

Parameters	Mean ± SD or *N* (%)
Number of patients	35
ECG	Normal
CLT	Normal
Age (and range; years)	51.17 ± 14.53 (20–74)
Gender (males/females)	20 : 15
Eyes (left/right)	19 : 16
IOP (and range; mmHg)	11.89 ± 2.87 (5.0–20)
BCVA (LogMAR, range)	LP–1.3

ECG: 12-lead electrocardiogram; CLT: clinical laboratory test results; BCVA: best-corrected visual acuity; IOP: intraocular pressure.

**Table 2:** Concentrations of resveratrol and its major metabolites in the tissues of eyes.

Species	Mean ± SD or *N* (%)		
	Conjunctiva (nmol/g)	Aqueous humor (nmol/L)	Vitreous humor (nmol/L)
Resveratrol	17.19 ± 15.32		
	(0.97–45.12)	Bod	Bod
	*n* = 8		

Resveratrol-3-O-glucuronide	2.32 ± 2.00	86.11 ± 30.76	
	(0.46–6.85)	(58.44–119.24)	Bod
	*n* = 8	*n* = 3	

Resveratrol-4′-O-glucuronide	2.12 ± 2.09	85.17 ± 56.73	
	(0.35–7.14)	(14.40–148.46)	Bod
	*n* = 9	*n* = 4	

Resveratrol-3-sulfate	22.31 ± 24.18	364.9 ± 523.94	62.95 ± 41.97
	(1.20–76.65)	(23.38–1503.76)	(22.75–128.20)
	*n* = 17	*n* = 7	*n* = 6

Note: values are the mean ± SD of the samples from 35 eyes. Range is in brackets. *N*, the number of samples which could be quantitated; Bod, below the limit of detection.

**Table 3:** Concentrations of resveratrol and its major metabolites in the serum of three participants.

Species	Concentration (nmol/L)			Mean ± SD or *N* (%)
	Patient 1	Patient 2	Patient 3	
Resveratrol	174.1	337.7	21.9	177.9
Resveratrol-3-O-glucuronide	54.5	162.9	481.7	233.0
Resveratrol-4′-O-glucuronide	44.6	155.4	456	218.7
Resveratrol-3-sulfate	257.6	2112	3539	1969.5

Note: the blood was drawn at about 2 hours after the final dose of resveratrol.
